# Additive effects of inhibiting both mTOR and glutamine metabolism on the arthritis in SKG mice

**DOI:** 10.1038/s41598-019-42932-1

**Published:** 2019-04-23

**Authors:** Yo Ueda, Jun Saegusa, Takaichi Okano, Sho Sendo, Hirotaka Yamada, Keisuke Nishimura, Akio Morinobu

**Affiliations:** Rheumatology and Clinical Immunology, Kobe University Graduate School of Medicine, Kobe, Japan

**Keywords:** Rheumatoid arthritis, Immunosuppression, Chronic inflammation, T-helper 17 cells

## Abstract

Glutamine metabolism and the mechanistic target of rapamycin (mTOR) pathway are activated cooperatively in the differentiation and activation of inflammatory immune cells. But the combined inhibition of both pathways was rarely investigated. This study investigated how inhibiting both glutamine metabolism with 6-diazo-5-oxo-L-norleucine (DON) and mTOR with rapamycin affects immune cells and the arthritis in a mouse model. We revealed that rapamycin and DON additively suppressed CD4^+^ T cell proliferation, and both of them inhibited Th17 cell differentiation. While DON inhibited the differentiation of dendritic cells and macrophages and facilitated that of Ly6G^+^ granulocytic (G)-MDSCs more strongly than did rapamycin, G-MDSCs treated with rapamycin but not DON suppressed CD4^+^ T cell proliferation *in vitro*. The combination of rapamycin and DON significantly suppressed the arthritis in SKG mice more strongly than did each monotherapy *in vivo*. The numbers of CD4^+^ T and Th17 cells in the spleen were lowest in mice treated with the combination therapy. Thus, combined treatment with rapamycin and DON additively ameliorated the arthritis in SKG mice, possibly by suppressing CD4^+^ T cell proliferation and Th17 differentiation. These results suggest the combination of rapamycin and DON may be a potential novel therapy for arthritis.

## Introduction

Rheumatoid arthritis (RA) is a chronic inflammatory disease characterized by infiltrations of various leukocyte subpopulations into the synovial space and the developing pannus in multiple joints, leading to inflammation, cartilage destruction, and bone erosion^1^.

The metabolism of immune cells changes according to their state of activation or differentiation; this is called metabolic reprogramming. Inflammatory immune cells such as effector lymphocytes (mainly Th1 and Th17 cells) and M1 macrophages exhibit a high uptake of nutrients for glycolysis and glutamine metabolism. In contrast, regulatory cells primarily rely on fatty acid oxidation^2^.

Since rapamycin was originally identified as a potent suppressor of T cell proliferation^3^, the mechanistic target of rapamycin (mTOR) pathway becomes known as one of the main signaling pathways upregulated during the differentiation and activation of inflammatory immune cells^2^. mTOR exists in two distinct complexes, mechanistic target of rapamycin complex 1 (mTORC1) and mTORC2. Cytokines, TCR engagement and co-stimulation, growth factors induce the activation of phosphatidyl inositol 3-kinase (PI3K). PI3K signaling induces the phosphorylation of Akt on Thr308 via PIP3 and on Ser473 via mTORC2. Akt phosphorylation leads to the activation of mTORC1 which induces the synthesis of proteins, lipids, and nucleotides via the activation of ribosomal protein S6 kinase (S6K) and 4E-binding protein 1 (4E-BP1). mTORC2 phosphorylates and activates Akt to control cellular metabolism, survival, and cytoskeletal organization. mTORC1 and mTORC2 are closely related and constitute a key metabolic signaling network that coordinates many metabolic processes characterized in growing or proliferating cells^4–6^. The involvement of mTOR and glutamine metabolism in the activation and differentiation of lymphocytes as well as in tumor proliferation is well documented^7^. Glutamine metabolism increases during the activation of CD4^+^ T cells and their differentiation toward Th1 and Th17 cells^8,9^. In addition, mTOR is activated in the differentiation of immature DCs and the polarization of M1 macrophages. Although glutamine metabolism is upregulated in the polarization of M1 macrophages, its influence on DC differentiation is unknown^4,10–12^.

Myeloid-derived suppressor cells (MDSCs) are a heterogeneous population of immature immunosuppressive cells. This population expands in response to pathologies such as tumor or inflammation, while the normal differentiation into other myeloid cells is disturbed^13^. In mice, MDSCs are identified by the markers CD11b and Gr1. MDSCs can be divided phenotypically into two subsets: 1) granulocytic (G-MDSCs) or polymorphonucleolar (PMN-MDSCs), defined as CD11b^+^ Ly6G^+^ Ly6C^int^ cells, and 2) monocytic (M-MDSCs), defined as CD11b^+^ Ly6G^−^ Ly6C^high^ cells. MDSCs have several mechanisms for suppressing immune functions. They suppress T cell responses by producing arginase 1 (Arg-1), inducible nitric oxide synthase (iNOS), reactive oxygen species (ROS), and TGFβ, and by upregulating PD-L1^14,15^.

MDSCs have been shown to have protective potential in mouse models of arthritis^16–21^, central nervous system autoimmune disease^22^, colitis^23^, acute kidney disease (AKI)^24^, and graft versus host disease (GVHD)^25–28^. We previously reported that MDSCs are expanded in the bone marrow (BM) and spleen of SKG arthritic mice^17^ and in the lungs of SKG mice with interstitial lung disease^29^. Rapamycin exerts a protective effect by increasing the proportion of MDSCs in mouse models of GVHD and acute kidney injury (AKI)^24–26^. The SKG mouse is a well-established genetic model with many of the features of RA, including chronic destructive arthritis with a Th17 phenotype^30,31^. We previously demonstrated that inhibiting glycolysis or glutamine metabolism is therapeutic for the arthritis in SKG mice^32,33^. However, the role of mTOR in SKG mice and the effect of the mTOR inhibitor rapamycin has not been investigated. The metabolic processes of MDSCs are also unknown.

mTORC1 activation is triggered by the intracellular concentration of amino acids. Various amino acids are transported into the cells in exchange for glutamine through amino acid transporters, indicating the importance of glutamine in determining the amino acid density^9^. Because of this close relation between mTOR and amino acids including glutamine, the effect of simultaneously inhibiting mTOR and glutamine metabolism in immune cells has rarely been investigated. In this study, we show that the mTOR inhibitor rapamycin and the glutamine antagonist 6-diazo-5-oxo-L-norleucine (DON), a glutamine analog that competitively inhibits the action of glutamine, act additively on immune cells and have a therapeutic effect on the arthritis in the SKG mouse model of RA.

## Results

### Rapamycin and DON additively inhibited CD4^+^ T cell proliferation

**Figure 1:**
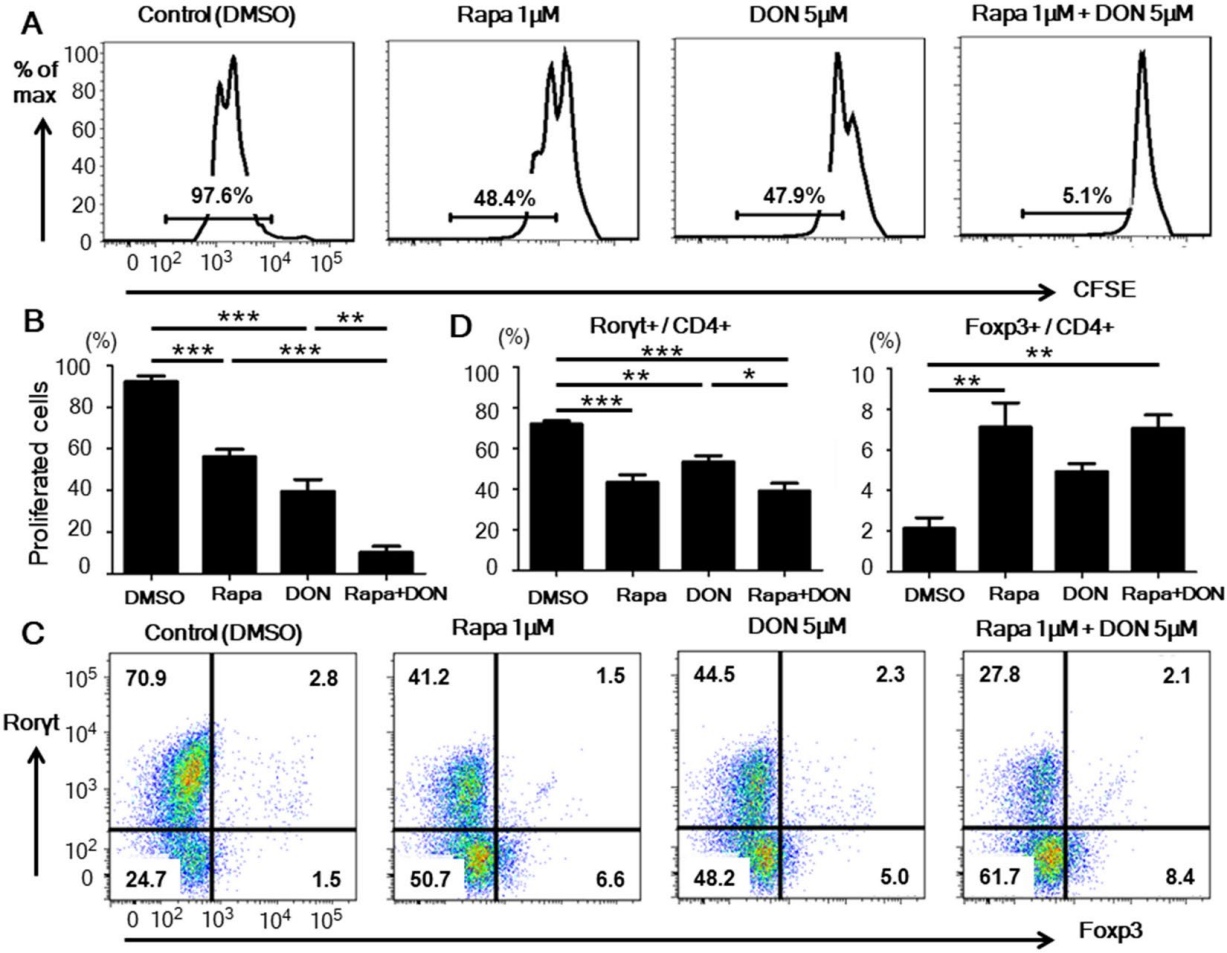
Rapamycin with DON additively suppressed CD4^+^ T cell proliferation *in vitro*. (**A**,**B**) CD4^+^ T cells, labeled with carboxyfluorescein diacetate succinimidyl ester (CFSE), were isolated from untreated Balb/c mice, placed in culture plates precoated with anti-CD3 and CD28 mAbs, and cultured with either DMSO (control), 1 μM rapamycin (Rapa), 5 μM DON, or the combination of 1 μM rapamycin and 5 μM DON (Rapa + DON). After 3 days of culture, the frequency of proliferating CD4^+^ T cells was assessed by measuring the CFSE fluorescence using flow cytometry. Data are representative of 4 independent experiments. (**C**,**D**) CD4^+^ T cells isolated from untreated Balb/c mice were placed in culture dishes precoated with anti-CD3 and anti-CD28 mAbs, and cultured in medium containing IL-17, TGF-β, anti-IFN-γ, anti-IL-4 antibodies, and one of the four drug regimens described in (**A**). The cells were collected on day 5 of culture and stained with anti-CD4, anti-Rorγt, and anti-Foxp3 antibodies, and the frequencies of Th17 (Rorγt^+^/CD4^+^) and Treg (Foxp3^+^/CD4^+^) cells were analyzed by flow cytometry. Data are representative of 4 independent experiments. Bars show mean ± SEM. ^*^P < 0.05; ^**^P < 0.01; ^***^P < 0.001.

We first investigated the effect of rapamycin, DON, or both on the proliferation of CD4^+^ T cells. CD4^+^ T cells stimulated with anti-CD3 and CD28 mAbs were cultured with one of four drug regimens: DMSO (control), 1 μM rapamycin, 5 μM DON, or 1 μM rapamycin plus 5 μM DON. Although treatment with rapamycin or DON alone significantly inhibited the proliferation of CD4^+^ T cells, treatment with rapamycin plus DON suppressed the CD4^+^ T cell proliferation even more strongly than either drug alone. (Fig. 1A,B) In addition, we confirmed a glutaminase 1 inhibitor, compound 968 (C968), also suppressed CD4^+^ T cell proliferation in a dose-dependent manner as a single drug and additively in combination with rapamycin as well as DON. (Suppl. Fig. 1), confirming the additive effects of glutamine metabolism and mTOR inhibition on CD4^+^ T cells. Although DON reduced the cell viability used as a single drug, adding rapamycin to the DON did not increase the proportion of dead cells compared to DON alone (Suppl Fig. 2). These results suggested that rapamycin and DON additively suppressed CD4^+^ T cell proliferation without increasing the cellular toxicity.

We studied phosphorylated status of Thr389 on S6K and Ser473 on Akt by Western blotting for assessing the activation of mTORC1 and mTORC2, respectively. CD4^+^ T cells stimulated with anti-CD3 and CD28 mAbs were cultured for 24 hrs with one of five drug regimens: DMSO, 1 μM rapamycin, 5 μM DON, 1 μM rapamycin plus 5 μM DON, or 0.1 μM Torin1 (an ATP-competing dual mTORC1 and mTORC2 inhibitor). We found that rapamycin with or without DON suppressed strongly both mTORC1 and mTORC2. On the other hand, DON suppressed the activation of mTORC1 partially and mTORC2 slightly. (Suppl. Fig. 3) In addition, the suppressive status of mTORC1 and mTORC2 by rapamycin is dependent on the concentration^34^. We studied the additive effects of various concentrations of rapamycin (0, 0.1, 1, 10, 100, and 1000 nM) in combination with 5 μM DON on CD4^+^ T cell proliferation. In the result, rapamycin more than 1 nM (which had been shown to inhibit both mTORC1 and mTORC2) in combination with DON significantly more suppressed CD4^+^ T cell proliferation compared to DON. On the other hand, the combination of 0.1 nM (which had been shown to inhibit mTORC1 selectively) rapamycin and DON showed slightly additive suppression compared to DON (not significant by One-way ANOVA with post-hoc analysis among all groups, but significant (p < 0.05) by direct comparison with paired t test). (Suppl. Fig. 4) Taken these results together, we suggested that the combination of rapamycin and DON was more additive when both mTORC1 and mTORC2 rather than only mTORC1 were suppressed by rapamycin.

### Rapamycin and DON inhibited the differentiation of Th17 cells

Metabolic change including mTOR pathway controls the balance of Th17 and Treg cells^35^. To determine how rapamycin and DON, alone or in combination, affect T cell differentiation, we added these drugs to cell cultures under Th17-promoting conditions. Treatment with rapamycin or DON significantly inhibited the differentiation of Rorγt^+^ Th17 cells, and combining the drugs did not produce an additive effect on Th17 differentiation (Fig. 1C,D). The proportion of Foxp3^+^ Treg cells was significantly higher in cells treated with rapamycin-including regimens compared to the other regimens (Fig. 1C,D). These results suggested that mTOR signaling is dominant to glutamine metabolism in controlling the Th17/Treg differentiation.

### Rapamycin and DON inhibited the BM cell differentiation into DCs and macrophages and promoted G-MDSC expansion *in vitro*

**Figure 2:**
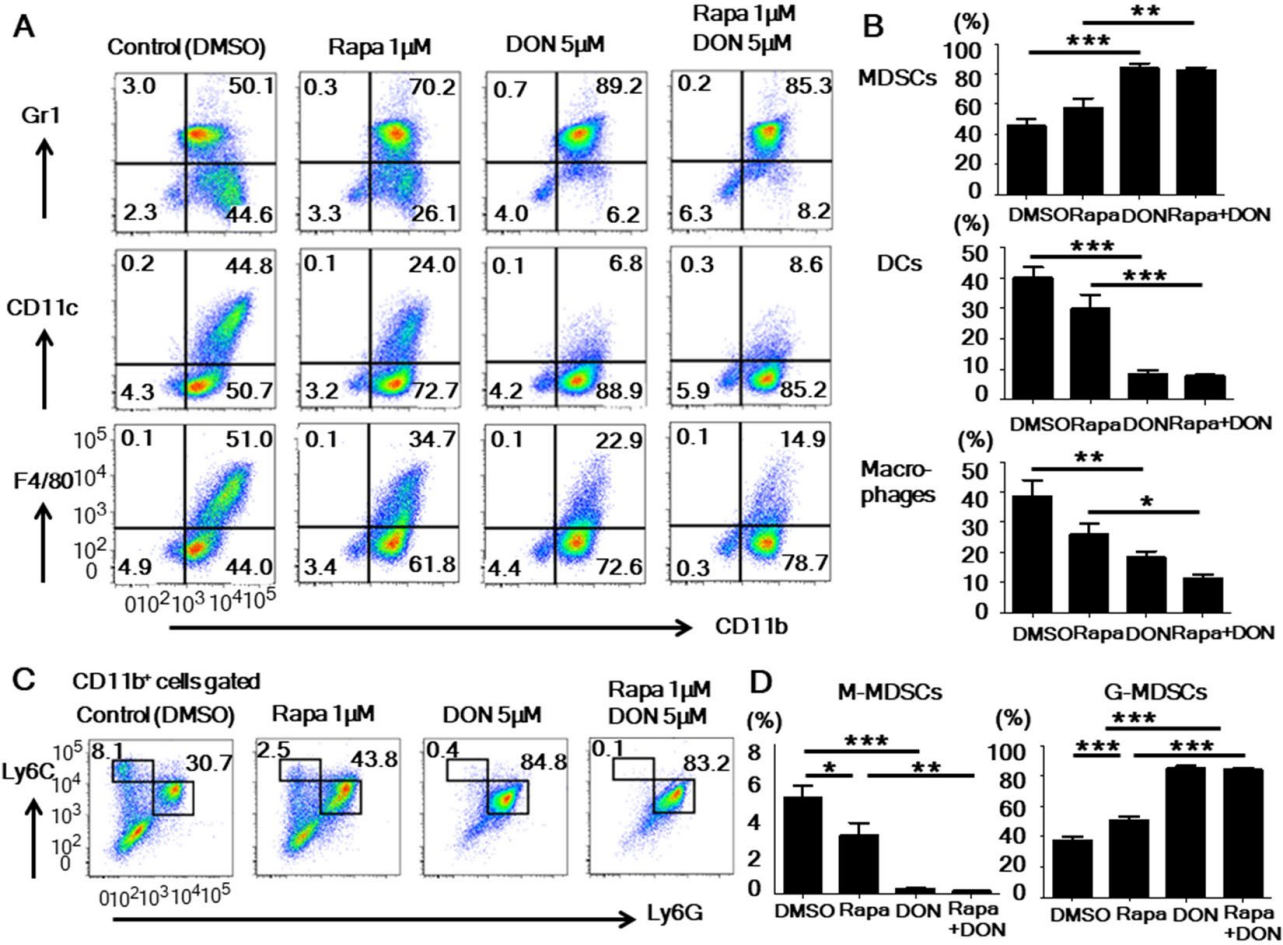
Rapamycin and DON inhibited the BM cell differentiation into DCs and macrophages and promoted G-MDSC expansion *in vitro*. (**A**,**B**) BM cells were stimulated with GM-CSF and cultured with the drug regimens described for lymphocyte culture in Fig. 1A. The cells were collected on day 5 and stained with anti-CD11b, anti-Gr1, anti-CD11c, and anti-F4/80 antibodies. The frequencies of DCs (CD11c^+^ CD11b^+^), macrophages (F4/80^+^ CD11b^+^), and MDSCs (Gr1^+^ CD11b^+^) were analyzed by flow cytometry. Data are representative of 4 independent experiments. (**C**,**D**) BM cells were collected on day 5 of culture and stained with anti-CD11b, anti-Ly6G, and anti-Ly6C antibodies. The frequency of M-MDSCs (CD11b^+^ Ly6G^−^ Ly6C^high^) and G-MDSCs (CD11b^+^ Ly6G^+^ Ly6C^int^) was analyzed by flow cytometry. Data are representative of 5 independent experiments. Bars show mean ± SEM. ^*^P < 0.05; ^**^P < 0.01; ^***^P < 0.001. Rapa, rapamycin.

Next, to examine how rapamycin and DON affect the differentiation of myeloid cells *in vitro*, we cultured BM cells stimulated with GM-CSF with the same four drug regimens used for the lymphocyte culture. When cultured with GM-CSF alone, the BM cells differentiated into DCs (CD11c^+^ CD11b^+^), macrophages (F4/80^+^ CD11b^+^), and MDSCs (Gr1^+^ CD11b^+^). DON significantly inhibited the differentiation of DCs and facilitated the expansion of MDSCs. The combined treatment with rapamycin and DON tended to inhibit the differentiation of macrophages more strongly than did rapamycin or DON alone (Fig. 2A,B). Both rapamycin and DON inhibited the differentiation of M-MDSCs (CD11b^+^ Ly6G^−^ Ly6C^high^) but facilitated the expansion of G-MDSCs (CD11b^+^ Ly6G^+^ Ly6C^int^) when these two MDSC subsets were analyzed separately (Fig. 2C,D). These results indicated that DON has a stronger effect than rapamycin in suppressing the DC, macrophage, and M-MDSC differentiation and promoting G-MDSC expansion.

### Rapamycin generated granulocytic MDSCs with stronger immunosuppressive ability *in vitro*

**Figure 3:**
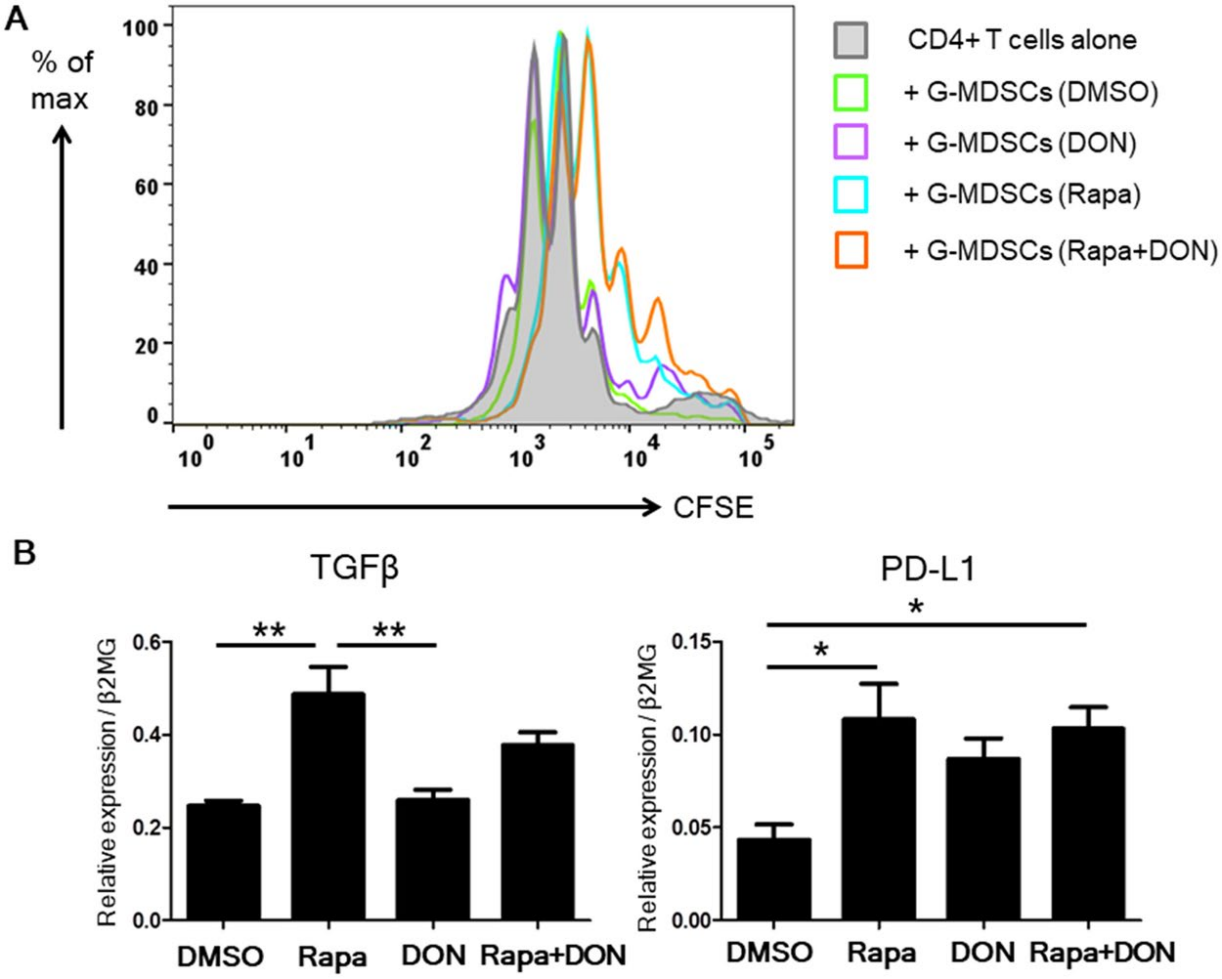
*In vitro–*generated G-MDSCs cultured with rapamycin had increased immunosuppressive ability. (**A**) *In vitro–*generated G-MDSCs were cultured with DMSO, 1 μM rapamycin (Rapa), 5 μM DON, or 1 μM rapamycin plus 5 μM DON (Rapa + DON), and were then isolated by manual MACS against Ly6G^+^ and their immunosuppressive ability assessed. CFSE-labeled CD4^+^ T cells were cultured alone or co-cultured at a 1:1 ratio with *in vitro*–generated G-MDSCs and stimulated by coating the culture plate with anti-CD3 and CD28 mAbs. After 3 days of culture, the proliferation of CD4^+^ T cells cultured alone or with G-MDSCs was examined by measuring the CFSE fluorescence using flow cytometry. Results shown are representative of four separate experiments. (**B**) Real-time PCR analysis of the TGFβ and PD-L1 mRNA levels in isolated *in vitro–*generated G-MDSCs cultured under the four drug regimens. Each experiment was performed in triplicate. Data are representative of 5 independent experiments. Bars show mean ± SEM. ^*^P < 0.05; ^**^P < 0.01.

We next focused on the function of G-MDSCs treated with rapamycin, DON, or both. We isolated *in vitro*–generated G-MDSCs and assessed their immunosuppressive ability. The purity of the isolated G-MDSCs was above 90% as assessed by flow cytometry (Suppl Fig. 5). Treating the G-MDSCs with rapamycin inhibited the proliferation of co-cultured CFSE-labeled CD4^+^ T cells (Fig. 3A). On the other hand, *in vitro–*generated G-MDSCs that were not treated with rapamycin had no remarkable immunosuppressive ability. Moreover, treating the *in vitro–*generated G-MDSCs with rapamycin increased their expression of TGF-β and PD-L1 (Fig. 3B). These results suggested that mTOR, but not glutamine metabolism, is related to the immunosuppressive ability of G-MDSCs.

### Rapamycin and DON additively ameliorated the arthritis in SKG mice

**Figure 4:**
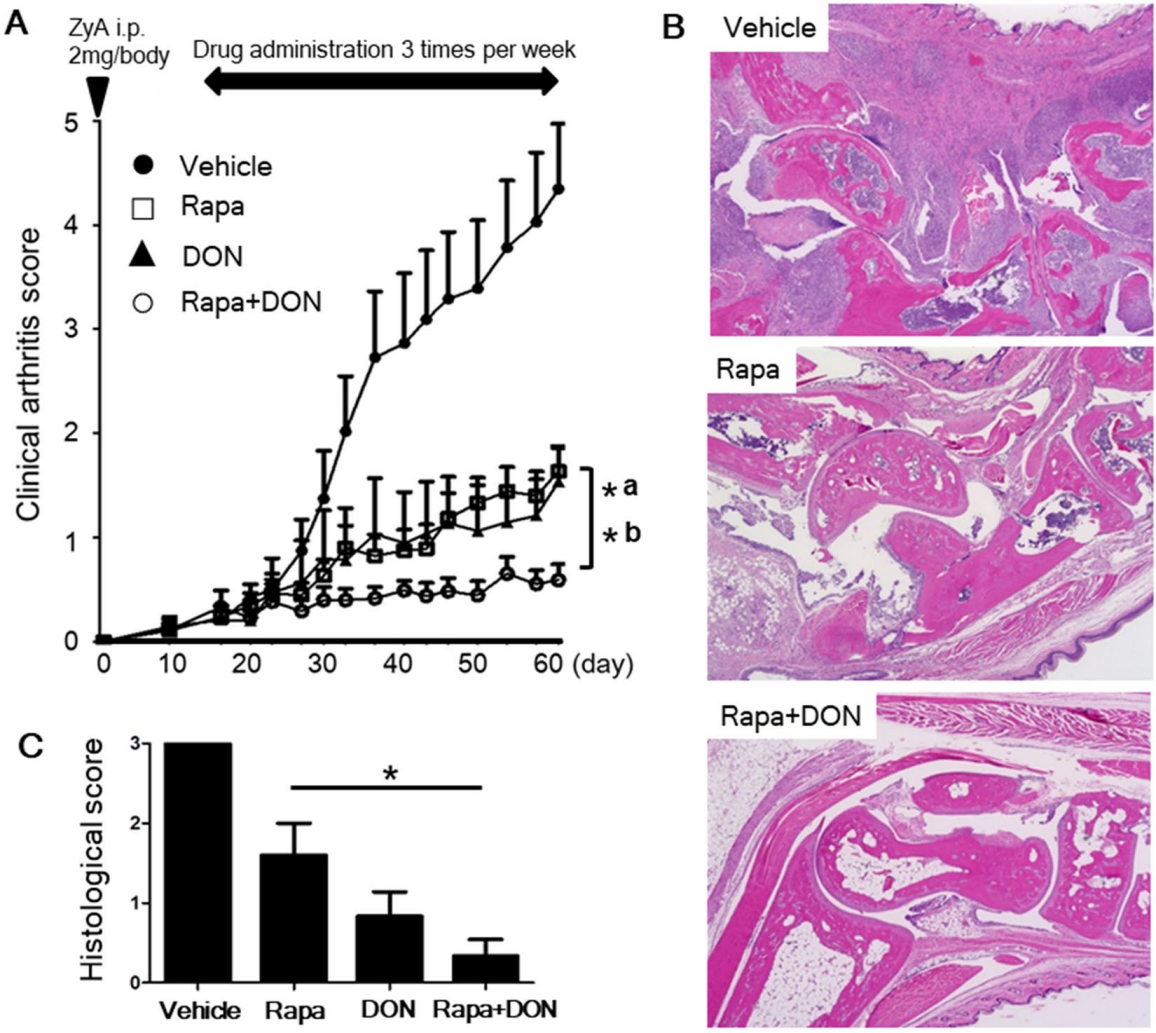
Rapamycin and DON additively ameliorated the arthritis in SKG mice. (**A**) SKG mice with ZyA-induced arthritis were given thrice-weekly intraperitoneal injections of vehicle (*n* = 11), 3 mg/kg rapamycin (Rapa, *n* = 12), 1.6 mg/kg DON (*n* = 11), or 3 mg/kg rapamycin plus 1.6 mg/kg DON (Rapa + DON; *n* = 13). We recorded clinical arthritis scores for the mice up to day 62 after the ZyA injection, and compared the final scores between mice treated by the various drugs regimens; bracket shows differences between mice treated with Rapa + DON and those treated by (a) Rapa or (b) DON alone. (**B**) The hind paws of SKG mice were evaluated for histopathological changes on day 62 after the ZyA injection. Specimens were stained with hematoxylin and eosin; original magnification 40×. (**C**) Histologic scores in the left hind paw for SKG mice treated with vehicle (*n* = 5), Rapa (*n* = 5), DON (*n* = 6), or Rapa + DON (*n* = 6). Bars show mean ± SEM. ^*^P < 0.05.

Finally, we examined the therapeutic effect of rapamycin and DON *in vivo*. Both rapamycin and DON significantly suppressed the arthritis in SKG mice, as assessed by clinical arthritis scores, and treatment with rapamycin and DON suppressed arthritis more strongly than did rapamycin or DON alone (Fig. 4A). Of the four drug regimens tested, the histological scores were lowest for the group treated with the combination of rapamycin and DON (Fig. 4B,C). All treatment regimens, including the combination of rapamycin and DON, were well tolerated, with no therapy-related deaths or loss of body weight (Suppl Fig. 6).

**Figure 5:**
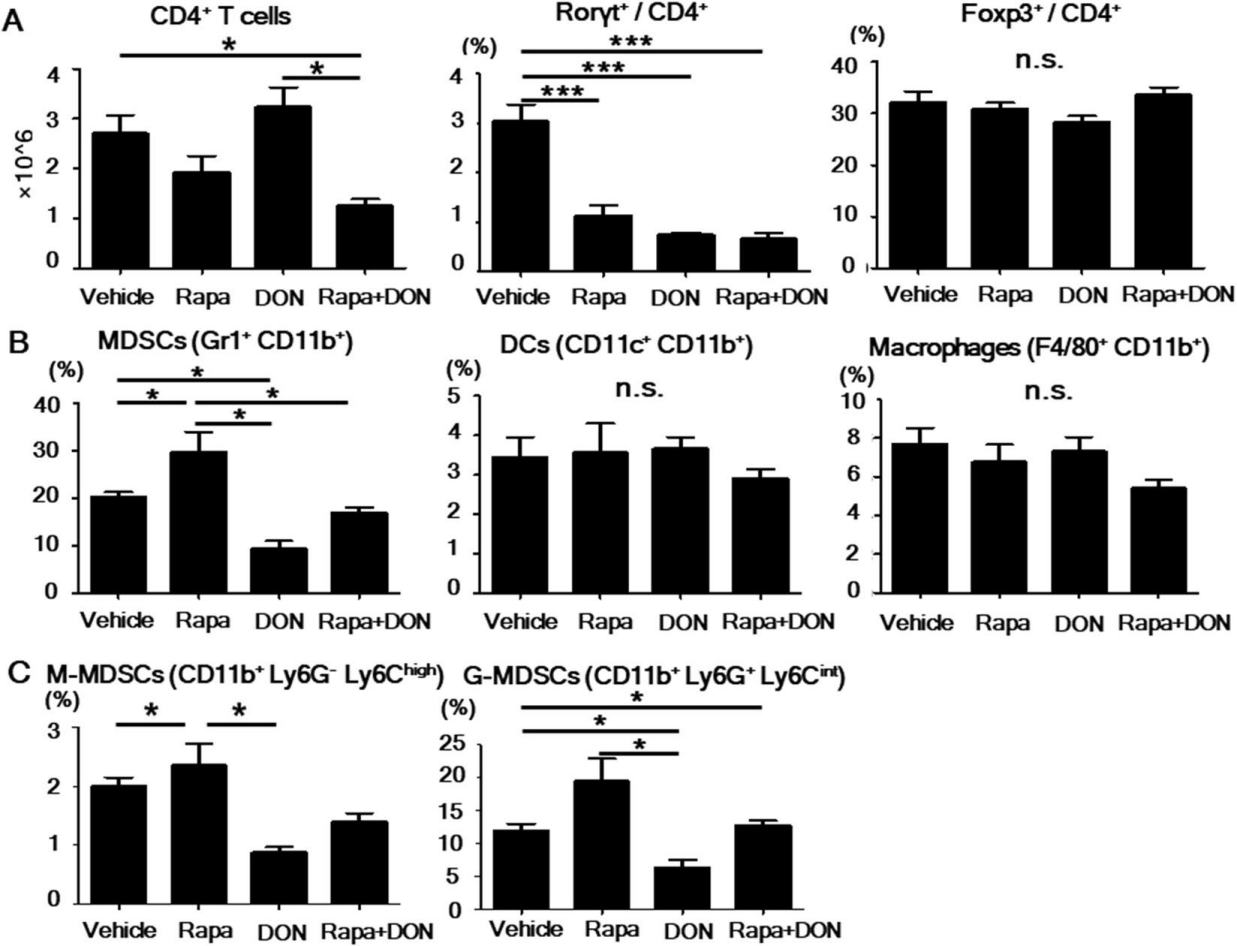
CD4^+^ T and Th17 cell counts in the spleen were lowest after the combined treatment with rapamycin and DON. (**A**) The frequency of CD4^+^ T, Th17 (Rorγt^+^/CD4^+^), and Treg (Foxp3^+^/CD4^+^) cells in splenocytes treated with various drug regimens was analyzed by flow cytometry, and the number of CD4^+^ T cells was calculated. (**B**) The frequency of DCs (CD11c^+^ CD11b^+^), macrophages (F4/80^+^ CD11b^+^), and MDSCs (Gr1^+^ CD11b^+^) in the spleen was analyzed by flow cytometry. (**C**) The frequency of M-MDSCs (CD11b^+^ Ly6G^−^ Ly6C^high^) and G-MDSCs (CD11b^+^ Ly6G^+^ Ly6C^int^) was analyzed by flow cytometry. Bars show mean ± SEM; *n* = 5 rapamycin (Rapa), 6 DON, and 6 Rapa + DON. ^*^P < 0.05; ^***^P < 0.001; n.s. = not significant.

In the spleen, the number of CD4^+^ T cells was significantly lower in mice treated with both rapamycin and DON than in those treated by vehicle or DON only (Fig. 5A), and the proportion of Rorγt^+^ Th17 cells was significantly lower in mice treated with rapamycin, DON, or both than in the vehicle-treated group. The four groups had similar proportions of Foxp3^+^ Treg cells, DCs, and macrophages (Fig. 5A,B). Rapamycin-treated mice had a higher proportion of total MDSCs (CD11b^+^ Gr1^+^) and G-MDSCs (CD11b^+^ Ly6G^+^ Ly6C^int^) than did the other groups, and the proportion of these cells was lower in the DON-treated group than in the others. Compared to these single effects, the combination of DON and rapamycin had intermediate effects on myeloid cells *in vivo* (Fig. 5B,C). Taken together, these results indicated that rapamycin and DON additively ameliorated arthritis by suppressing CD4^+^ T cell proliferation and Th17 differentiation *in vivo*.

In the immunohistochemistry analysis of the paws of SKG mice treated with each drug regimen, we measured the presence of synoviocytes (stained with cadherin-11), myeloid cells (stained with CD11b), and T lymphocyte (stained with CD3). We assessed the extents of the presence of these cell populations by using semi-quantitative assessment (SQA) reported previously^36–38^. In the result, the extents of the presence of all three kinds of cell populations showed similar tendencies with clinical arthritis score, but the difference among Rapa, DON, and Rapa + DON groups was not shown. (Suppl. Fig. 7) From these results, although we couldn’t elucidate the target of additive effects of rapamycin and DON, we speculated both rapamycin and DON might affect synoviocytes as well as immune cells.

## Discussion

Our study demonstrated for the first time that the combination of rapamycin and DON had additive effects on immune cells *in vitro* and *in vivo*. We revealed that rapamycin and DON additively suppressed CD4^+^ T cell proliferation, and both of them inhibited Th17 cell differentiation. In myeloid cells, DON strongly inhibited DC and macrophage differentiation but facilitated the differentiation of G-MDSCs, while rapamycin enhanced the immunosuppressive ability of G-MDSCs and mildly suppressed DC and macrophage differentiation. Thus, rapamycin and DON additively induced the expansion and immunosuppressive effect of G-MDSCs. Finally, we demonstrated that the combined treatment with rapamycin and DON significantly suppressed arthritis in SKG mice compared to rapamycin or DON monotherapy.

Our study is also the first to demonstrate that inhibiting both mTOR and glutamine metabolism had an additive effect on CD4^+^ T cells. Activating naïve T cells with anti-CD3 and CD28 mAbs causes a rapid increase in glutamine uptake that is dependent on the amino acid transporters such as ASCT2 (SLC1A5) and LAT1^39,40^. Glutamine uptake also positively regulates differentiation into Th17 and Th1 cells^40–42^. Inhibiting mTORC1, which plays a significant role in activating CD4^+^ T cells and differentiating Th1 and Th17 cells, with rapamycin decreased the differentiation of Th1 and Th17 cells^43,44^. Although rapamycin could enhance the generation of induced Treg cells^45–47^, inhibition of both mTORC1 and mTORC2 was required for the generation of Treg cells in the absence of exogenous TGF-β in a previous report^34^. Thus, in addition to mTOR, glutamine and other amino acids are also important in activating CD4^+^ T cells. These two pathways seem to be closely connected, since amino acid inhibition regulates mTOR activation. In our study, the combination of rapamycin and DON additively suppressed CD4^+^ T cell proliferation. However, although both rapamycin and DON suppressed Th17 cell differentiation, the combination of DON and rapamycin did not have an additive effect. In addition, rapamycin but not DON promoted Treg cell differentiation *in vitro*. These results suggested that rapamycin and DON have similar but not identical effects on CD4^+^ T cell activation and differentiation. Prolonged treatment of rapamycin *in vitro* (more than 24 hrs) and *in vivo* (up to 6 weeks) showed the suppression of mTORC2 as well as mTORC1^48,49^. Although immunologic functions of mTORC2 are less understood rather than mTORC1, we assumed rapamycin suppressed both mTORC1 and mTORC2 *in vivo* judging from the treatment period (almost 6 weeks). In the Western blotting analysis of the proliferating CD4^+^ T cells stimulated for 24 hrs with anti-CD3 and CD28 mAbs, we found that 1 μM rapamycin suppressed strongly both mTORC1 and mTORC2, but 5 μM DON suppressed mTORC1 partially and mTORC2 slightly. It has been reported that in ASCT2 (a glutamine transporter) deficient CD4^+^ T cells, TCR and CD28-mediated mTORC1 activation was severely attenuated and recovered by an excessive amount of glutamine, while the activation of mTORC2 was not affected^39^. The difference of mTORC2 suppression between rapamycin and DON could explain the difference of Treg differentiation *in vitro*. In addition, among various concentrations of rapamycin (0, 0.1, 1, 10, 100, and 1000 nM), rapamycin more than 1 nM (which had been shown to inhibit both mTORC1 and mTORC2) plus DON significantly more suppressed CD4^+^ T cell proliferation compared to DON, but 0.1 nM (which had been shown to inhibit mTORC1 selectively) rapamycin plus DON showed slightly additive suppression compared to DON *in vitro*. From these results, we speculated that DON affected CD4^+^ T cells through the suppression of mTOR, dominantly mTORC1, but also through mTOR-independent mechanisms which possibly contributed to the additive effect on CD4^+^ T cell proliferation.

We showed that DON had a stronger effect than rapamycin on inhibiting the differentiation of DCs and macrophages and facilitating the differentiation of MDSCs, suggesting that glutamine metabolism is essential for both DC and macrophage differentiation. The mechanism by which glutamine metabolism influences the differentiation of DCs is not clear; however, mTOR is upregulated in the differentiation of DCs and M1 macrophages^4,10–12^.

Although the metabolism of MDSCs has not been clarified, several studies have reported on the influence of rapamycin on MDSCs. Rapamycin prolongs graft survival and ameliorates AKI by inducing MDSCs, primarily G-MDSCs^24–26^. mTOR is reported to govern M-MDSCs in mouse allograft and tumor models, and glycolysis via mTOR activation is essential for M-MDSCs to differentiate and acquire immunosuppressive ability^25^. The same study found that the proportion of G-MDSCs was not influenced by an mTOR inhibitor or a cell-specific mTOR knockout. We found that both DON and rapamycin inhibited the differentiation of M-MDSCs and facilitated the differentiation of G-MDSCs. Intriguingly, DON had stronger effects on myeloid cell differentiation than on G-MDSC differentiation. Thus, glutamine metabolism may be essential for the differentiation of DCs, macrophages, and M-MDSCs, but not G-MDSCs.

We showed that treating *in vitro*–generated G-MDSCs with rapamycin increased their immunosuppressive effect and enhanced their expression of TGF-β and PD-L1. Rapamycin has been shown to upregulate immunosuppressive ability in both MDSC subtypes^24,26^. The adoptive transfer of rapamycin-treated MDSCs upregulated serum TGF-β1 levels in an AKI model^24^. In an experimental autoimmune encephalomyelitis, elevated PD-L1 expression mediated the protective effects of MDSCs, whereas no Arg-1 or NO was detected in G-MDSCs^22^. Likewise, Arg-1, iNOS, and ROS were not upregulated in rapamycin-treated MDSCs in our study. Taken together with previous studies, our results indicate that rapamycin facilitates the differentiation of G-MDSCs and promotes their immunosuppressive ability, possibly by upregulating PD-L1 expression and TGF-β production. Given that DON doesn’t increase the immunosuppressive ability of G-MDSCs, rapamycin’s effect on G-MDSCs may be independent of glutamine metabolism.

We demonstrated that the inhibitors rapamycin and DON had additive and therapeutic effects in an arthritis mouse model. Several reports describe strategies that simultaneously inhibit two or more metabolic pathways. Simultaneously inhibiting mTOR and glutamine metabolism suppressed brain tumor proliferation^50^. A triple-therapy regimen consisting of DON, the glycolytic inhibitor 2-DG, and metformin was effective for GVHD by suppressing CD4^+^ and CD8^+^ effector T cells^51^. Metformin is a commonly used oral hypoglycemic drug that inhibits mTOR and mitochondrial respiratory complex I, activates AMPK, and promotes fatty acid oxidation. This triple therapy profoundly suppressed activation of the mTOR pathway, as assessed by phosphorylated S6K. We at first thought that rapamycin and DON suppressed the same metabolic pathway, and that DON might inhibit mTOR signaling by reducing the influx of amino acids. However, the suppression of mTOR by DON was weaker than rapamycin in the proliferating CD4^+^ T cells. Thus, we speculate that the additive therapeutic effects we observed in the SKG arthritis model might be explained by the inhibition of glutamine metabolism at the same time that mTOR signaling and mTOR-induced glycolysis were also inhibited, but further investigation is necessary to determine the metabolic profiles involved.

Our *in vitro* and *in vivo* findings agree in some but not all points. Rapamycin and DON additively inhibited CD4^+^ T cell proliferation; when administered separately, rapamycin and DON inhibited Th17 differentiation both *in vitro* and *in vivo*. This point of agreement between the *in vitro* and *in vivo* results suggested that the additive effect of rapamycin and DON acts primarily on lymphocytes in SKG mice.

On the other hand, rapamycin-treated mice had higher proportions of total MDSCs and G-MDSCs than did the other groups, including those treated with both DON and rapamycin *in vivo*, while treatment with both rapamycin and DON promoted G-MDSC differentiation *in vitro*. Since MDSCs are induced by inflammatory cytokines or mediators in arthritic mice^16^, this point of discord might reflect residual inflammation in the rapamycin-treated group. Another possible explanation for this discord is the dosage of DON. DON decreased Th17 /CD4^+^ T cell ratio both *in vitro* and *in vivo*, but did not decrease CD4^+^ T cell counts *in vivo* in our series of experiments, suggesting that the dose of DON we used *in vivo* was lower than that of *in vitro* experiments.

In this study, DON significantly suppressed arthritis even when administered alone. As a glutamine analog, DON acts as an inhibitor of various glutamine-utilizing enzymes involved in several important metabolic pathways, such as purine, pyrimidine, and amino acid synthesis as well as glutaminase in the first step of glutamine metabolism. We confirmed a glutaminase 1 inhibitor, compound 968 (C968), also suppressed CD4^+^ T cell proliferation similarly with DON, indicating the crucial role for glutamine metabolism. (Suppl. Fig. 1) We have previously reported that C968 suppressed the proliferation of fibroblast-like synoviocytes derived from RA patients (RA-FLS) and ameliorated clinical arthritis in SKG mice^33^. We have found that DON also suppressed the proliferation of RA-FLS in a dose-dependent manner (data not shown). In the immunohistochemistry of the hind paw, the extents of the proliferation of synoviocytes as well as myeloid cells and T lymphocytes were suppressed in Rapa, DON, and Rapa + DON groups. Thus, we speculate that both rapamycin and DON attenuates arthritis by directly suppressing synovial cell proliferation as well as affecting immune cells in SKG mice. When DON was tested in clinical trials for cancer in the 1980s, researchers reported that it had severe dose-limiting toxicity with nausea and vomiting and without any obvious efficacy as a monotherapy^50^. In recent years, however, recognition of the importance of glutamine metabolism and glycolysis in cancer has spurred new interest in therapies that inhibit several metabolic pathways simultaneously^51–53^. In the present study, adding rapamycin to DON did not increase the proportion of dead cells *in vitro*, and no toxicity was observed for the combination therapy *in vivo*, suggesting that these drugs might be clinically feasible.

In summary, combined treatment with rapamycin and DON additively ameliorated arthritis in SKG mice, mainly by suppressing CD4^+^ T cell proliferation and Th17 differentiation. The simultaneous inhibition of mTOR and glutamine metabolism may represent a novel therapeutic strategy for RA.

## Materials and Methods

### Ethical provisions

This study was approved by the President of Kobe University after the review by Institutional Animal Care and Use Committee (Permission number: P 170601) and carried out according to the Kobe University Animal Experimentation Regulations.

### Animals

Female SKG and BALB/c mice were obtained from CLEA Japan, Inc. The mice were kept at the Kobe University animal facility at a constant temperature with unrestricted access to laboratory chow and water. All procedures were conducted according to the recommendations of the Institutional Animal Care Committee of Kobe University.

### Reagents and antibodies

Zymosan A (ZyA; Alfa Aesar), DMSO (Tocris Bioscience), Rapamycin (Adipogen), 6-Diazo-5-oxo-L-norleucine (DON; Sigma-Aldrich), Torin1 (Tocris Bioscience), compound 968 (C968; Calbiochem) were purchased. *In vitro* experiments used the carboxyfluorescein diacetate succinimidyl ester (CFSE) cell proliferation kit (Invitrogen), RPMI 1640 (Wako Pure Chemical Industries), fetal bovine serum (FBS; MP Biomedicals), 1% penicillin-streptomycin (Lonza Walkersville), and recombinant murine granulocyte-macrophage colony-stimulating factor (GM-CSF; PeproTech). For flow cytometry analysis of myeloid cells, we used FITC-conjugated anti-Gr1 (BD PharMingen), FITC-conjugated anti-Ly6G (BD PharMingen), PerCP-conjugated anti-CD11b (BioLegend), APC-conjugated anti-Ly6C (BioLegend), APC-conjugated anti-CD11c (eBioscience), and PE-conjugated anti-F4/80 (eBioscience). For the flow cytometry analysis of lymphocytes, we used FITC-conjugated anti-CD4 (eBioscience), APC-conjugated anti-Rorγt (eBioscience), PE-conjugated anti-Foxp3 (eBioscience), and 7-AAD Viability Staining Solution (eBioscience). For the Western blotting analysis, anti-phospho-p70 S6 kinase (Thr389) (Cell Signaling, #97596 S), anti-p70 S6 kinase (Cell Signaling, #9202), anti-phospho-Akt (Ser473) (Cell Signaling, #4060), anti-Akt (Cell Signaling, #2920), and anti-β-actin antibodies (Sigma-Aldrich) were purchased.

### Flow cytometry

For phenotypical analysis, cells were washed with Flow Cytometry Staining Buffer (BioLegend) and stained with anti-Gr1, anti-Ly6G, anti-Ly6C, anti-CD11b, anti-CD11c, anti-F4/80, and anti-CD4 mAbs for 30 minutes at 4 °C. For the intracellular staining of CD4^+^ T cells, cultured CD4^+^ T cells stained with an anti-CD4 mAb were treated with the Intracellular Fixation & Permeabilization Buffer Set (BioLegend). These cells were analyzed on a FACS Verse (BD Bioscience) using FlowJo software (Tree Star).

### Generation of adherent cells from BM progenitors

BM cells (2 × 10^6^) from SKG mice were cultured in 2 ml RPMI 1640 medium supplemented with 10% FBS, 1% penicillin-streptomycin, 10 ng/ml GM-CSF, 50 μM 2-ME, and one of four drug regimens: 1 μM rapamycin (Rapa), 5 μM DON, the combination of 1 μM rapamycin and 5 μM DON (Rapa + DON), or DMSO (control). The cultures were maintained in 12-well plates at 37 °C in a 5% CO2-humidified atmosphere. On day 3 of culture, floating cells were gently removed and the medium was replaced with fresh medium with the same dose of GM-CSF and the same drugs. On day 5, the cells were collected and analyzed by flow cytometry.

### Isolation of G-MDSCs and CD4^+^ T cells

To isolate G-MDSCs from the single-cell suspensions of cultured BM cells from Balb/c mice, we used a biotinylated mAb against Ly6G and streptavidin-coated magnetic beads (Miltenyi Biotec) according to the manufacturer’s protocol. To isolate CD4^+^ T cells from single-cell suspensions prepared from Balb/c splenocytes, we used a biotinylated mAb against CD4, streptavidin-coated magnetic beads, and a manual MACS system (all from Miltenyi Biotec) according to the manufacturer’s protocol.

### CD4^+^ T cell proliferation

Isolated CD4^+^ T cells were incubated with 10 μM CFSE according to the manufacturer’s protocol, and then suspended in RPMI 1640 medium containing 10% FBS and 1% penicillin-streptomycin. For the co-culture analysis, CFSE-labeled CD4^+^ T cells (1 × 10^5^ in 100 μl of medium) were cultured in a 96-well flat-bottomed plate with equal numbers of *in vitro*–generated Ly6G^+^ cells cultured with the control, rapamycin, DON, or Rapa + DON regimen. To assess the direct influence of these drug regimens, CFSE-labeled CD4^+^ T cells (2 × 10^5^ in 200 μl of medium) were cultured with each drug condition for 3 days in 96-well flat-bottomed plates precoated with 10 μg/ml anti-CD3 mAb and 5 μg/ml anti-CD28 mAb. In some experiments, various concentrations of DON and C968 with or without 1 μM rapamycin were administered. CD4^+^ T cell proliferation was determined by measuring the CFSE fluorescence using flow cytometry. *In vitro–*generated lymphocyte viability was assessed by measuring the stained 7-AAD fluorescence using flow cytometry.

### Th17 cell differentiation

CD4^+^ T cells isolated by manual MACS were cultured for 5 days at 37 °C under Th17 conditions: the plates were precoated with 10 ng/ml anti-CD3 and 5 ng/ml anti-CD28 mAbs, and the medium was supplemented with 20 ng/ml IL-6, 5 ng/ml TGF-β, 2.5 μg/ml anti-IFN-γ mAb, and 2.5 μg/ml anti-IL-4 mAb. DMSO (control), 1 μM rapamycin, 5 μM DON, or the combination of 1 μM rapamycin and 5 μM DON was added to the culture medium. On day 3 of culture, half of the medium was replaced with fresh medium containing the same dose of cytokines and antibodies and a half-dose of the drug regimen. The cells were collected on day 5 and analyzed by flow cytometry.

### Reverse transcription-quantitative polymerase chain reaction (RT-qPCR)

Total RNA was isolated using RNeasy (Qiagen), and 1 μg of total RNA was reverse transcribed with a QuantiTect reverse transcription kit (Qiagen). RT-qPCR was performed using a QuantiTect SYBR Green PCR Kit (Qiagen) with an ABI Prism 9900 (Applied Biosystems) according to the manufacturers’ instructions. The primers used in the present study (listed in Supplemental Table 1) were purchased from Qiagen. The mRNA levels were normalized to that of β2 microglobulin (β2MG).

### Western blotting

Isolated CD4^+^ T cells stimulated with anti-CD3 and CD28 mAbs were cultured for 24 hrs with one of five drug regimens: DMSO, 1 μM rapamycin, 5 μM DON, 1 μM rapamycin plus 5 μM DON, or 0.1 μM Torin1. Cell lysates were analyzed by Western blotting with anti-S6 kinase (S6K), anti-phospho-S6 kinase (Thr389), anti-Akt, anti-phospho-Akt (Ser473), and anti-β-actin antibodies. The bound antibodies were visualized using a chemiluminescence reagent (Super Signal West Dura Extended Duration Substrate, Thermo Fisher Scientific) following the manufacturer’s instructions.

### Arthritis induction

Eight-week-old SKG mice were treated with 2 mg ZyA, as previously described^30^. Briefly, ZyA suspended in distilled water was injected intraperitoneally on day 0. Arthritis developed between 14 and 21 days after injection.

### Rapamycin and DON treatment of SKG mice

Rapamycin was dissolved in DMSO. DON was dissolved in distilled water. Beginning two weeks after the ZyA injection, the mice were given intraperitoneal injections three times a week of vehicle, 3 mg/kg rapamycin, 1.6 mg/kg DON, or 3 mg/kg rapamycin plus 1.6 mg/kg DON. The drugs were diluted to a total amount of 150 μl/body with PBS containing the same amount of DMSO. *In vivo* experiments, including experiments with the four drug regimens, were performed twice in separate experiments. Analysis of the spleen was performed in one experiment.

### Evaluation of arthritis

The development and severity of arthritis were assessed using a previously described scoring system^18,54^, as follows: 0 = no joint swelling, 0.1 = swelling of one digit joint, 0.5 = mild swelling of the wrist or ankle, and 1.0 = severe swelling of the wrist or ankle. The scores were totaled for each mouse, for a maximum possible clinical arthritis score of 5.8.

### Histology

The hind paws were removed, fixed in 4% paraformaldehyde, decalcified in EDTA, embedded in paraffin, sectioned, and stained with hematoxylin and eosin. The specimens were evaluated using a previously described histologic scoring system^17,54^, in which 0 = no inflammation; 1 = slight thickening of the synovial cell layer and/or some inflammatory cells in the sublining; 2 = thickening of the synovial lining, infiltration of the sublining, and localized cartilage erosion; and 3 = infiltration into the synovial space, pannus formation, cartilage destruction, and bone erosion.

### Immunohistochemistry

Paraffin-embedded tissue sections (5 μm) were deparaffinized and hydrated with xylene and graded alcohols using a standard protocol, and incubated overnight with primary antibodies at 4 °C in a humidified chamber, and then rinsed and incubated with biotinylated secondary antibodies for 30 min at room temperature. The slides were developed using the ImmunoCruz™ ABC Staining System (Santa Cruz Biotechnology) and were counterstained with Mayer’s hematoxylin solution (Wako). Immunohistochemical staining with anti-cadherin-11 (Abcam, # ab151302), anti-CD11b (Abcam, #ab133357), and anti-CD3 (Abcam, #ab16669) antibodies was performed. Assessment of the extents of cell populations were performed by using semi-quantitative assessment (SQA) reported previously^36–38^. In summary, SQA was performed in three high power fields (HPFs; ×400) in each sample by two independent observers. The expression of cadherin-11, CD11b, and CD3 was scored on a 5-point scale (0–4; score 0 represented minimal infiltration, while score 4 represented infiltration by numerous cells) and the mean of the three scores was calculated in each sample.

### Statistical analysis

Results were expressed as the mean ± SEM. Statistical analysis was performed by one-way ANOVA with post-hoc analysis for comparison among multiple groups. *P* values less than 0.05 were considered statistically significant.

## Supplementary information

Supplementary figures and table
