# Supplementary material for: Additive effects of inhibiting both mTOR and glutamine metabolism on the arthritis in SKG mice
Source: Sci Rep. 2019 Apr 23;9:6374. doi: 10.1038/s41598-019-42932-1 (PMC6476881; doi:10.1038/s41598-019-42932-1)
Supplement: Supplementary file 1 — Supplementary figures and table [file 41598_2019_42932_MOESM1_ESM.pdf]

Additive effects of inhibiting both mTOR and glutamine metabolism on the arthritis in SKG mice

Yo Ueda,[1] M.D., Jun Saegusa,[1] M.D., Ph.D., Takaichi Okano,[1] MD, Ph.D., Sho Sendo,[1] M.D., Ph.D., Hirotaka Yamada,[1] M.D., Keisuke Nishimura,[1] MD, Ph.D., and Akio Morinobu,[1] MD, Ph.D.

[1]Rheumatology and Clinical Immunology, Kobe University Graduate School of Medicine, Kobe, Japan.

Corresponding author: Akio Morinobu

7-5-1 Kusunoki-Cho, Chuo-Ku, Kobe 650-0017, Japan

Phone +81-78-382-6197, Fax +81-78-382-6209

Email: morinobu@med.kobe-u.ac.jp

Conflicts of interest: none

Suppl Table 1. Primers used in this study

Gene		Primer sequences (5′-3′)
**β2-microglobulin**	Forward primer	TTCTGGTGCTTGTCTCACTGA
	Reverse primer	CAGTATGTTCGGCTTCCCATTC
**TGFβ**	Forward primer	GCTAATGGTGGACCGCAACAAC
	Reverse primer	GCACTGCTTCCCGAATGTCTG
**PD-L1**	Forward primer	TGCTTCTCAATGTGACC
	Reverse primer	GGAACAACAGGATGGAT
**Arg-1**	Forward primer	CCAGAAGAATGGAAGAGTCAGTGT
	Reverse primer	GCAGATATGCAGGGAGTCACC
**iNOS**	Forward primer	CACCAAGCTGAACTTGAGCG
	Reverse primer	CGTGGCTTTGGGCTCCTC
**NOX2**	Forward primer	TGTGGTTGGGGCTGAATGTC
	Reverse primer	CTGAGAAAGGAGAGCAGATTTCG

Suppl Figure 1. CFSE-labeled $CD4^+$ T cells isolated from untreated Balb/c mice were cultured with several concentrations of compound 968 (C968; an inhibiter of glutaminase 1) and DON as a single drug (A) and in combination with 1 μM of rapamycin (B). After 3 days of culture, the collected cells were assessed the CFSE fluorescence using flow cytometry.

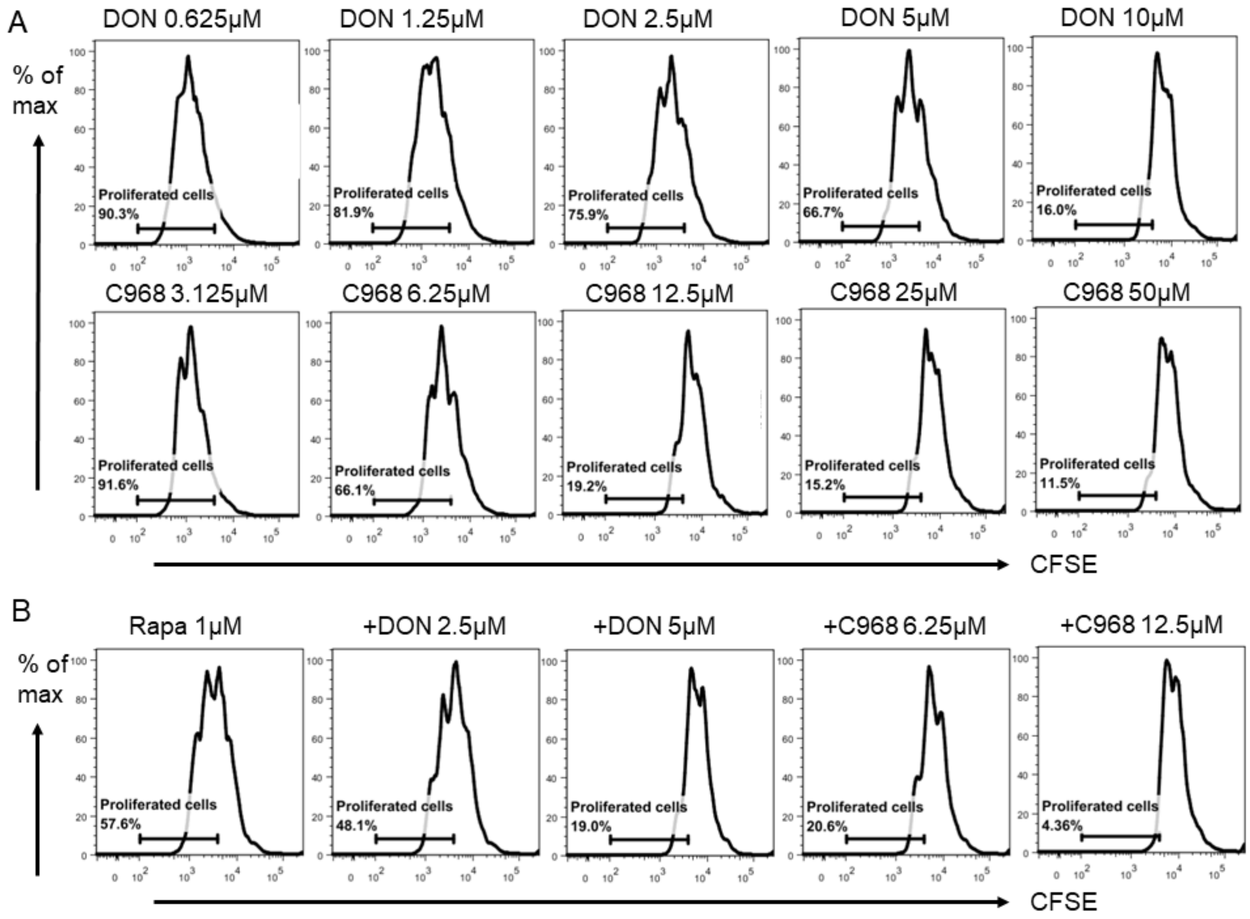

Suppl Figure 2. Viability of CD4+ T cells cultured with DSMO, rapamycin, DON, or rapamycin and DON were assessed by 7-AAD staining. CD4+ T cells isolated from untreated Balb/c mice were cultured as described in Figure 1A, B. After 3 days of culture, the collected cells were stained with 7-AAD, and the frequency of dead cells was determined by measuring the 7-AAD fluorescence using flow cytometry.

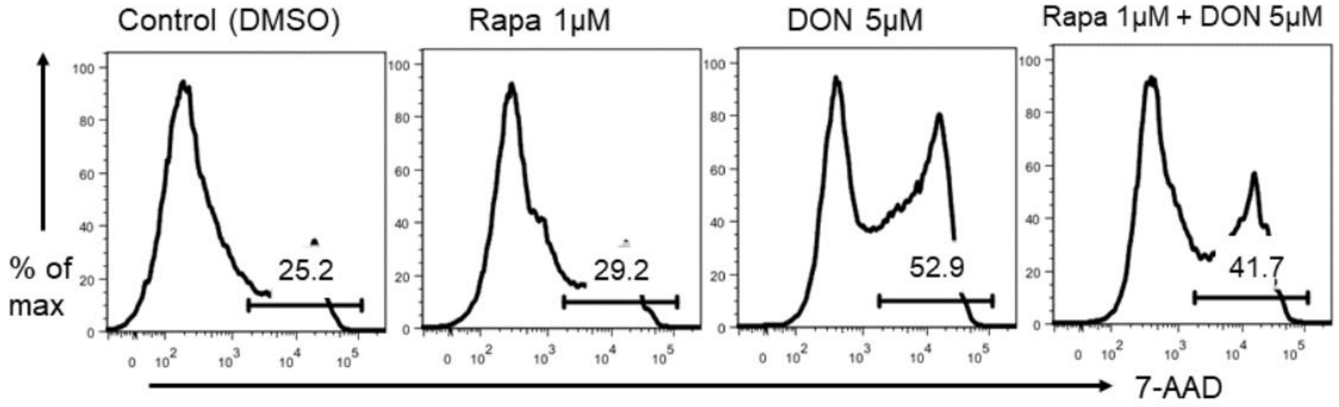

Suppl Figure 3. CD4+ T cells were cultured for 24hr in 96-well flat-bottomed plates precoated with 10 μg/ml anti-CD3 mAb and 5 μg/ml anti-CD28 mAb. DMSO, 0.1μM Torin1 (an ATP-competing dual mTORC1 and mTORC2 inhibiter), 1μM rapamycin (Rapa), 5μM DON (DON), or the combination of 1μM rapamycin and 5μM DON (Rapa+DON) were administered. Cell lysates were analyzed by Western blotting with anti-phospho-S6 kinase (Thr389), anti-S6 kinase (S6K), anti-phospho-Akt (Ser473), anti-Akt, and anti-β-actin antibodies. The bound antibodies were visualized using a chemiluminescence reagent following the manufacturer's instructions.

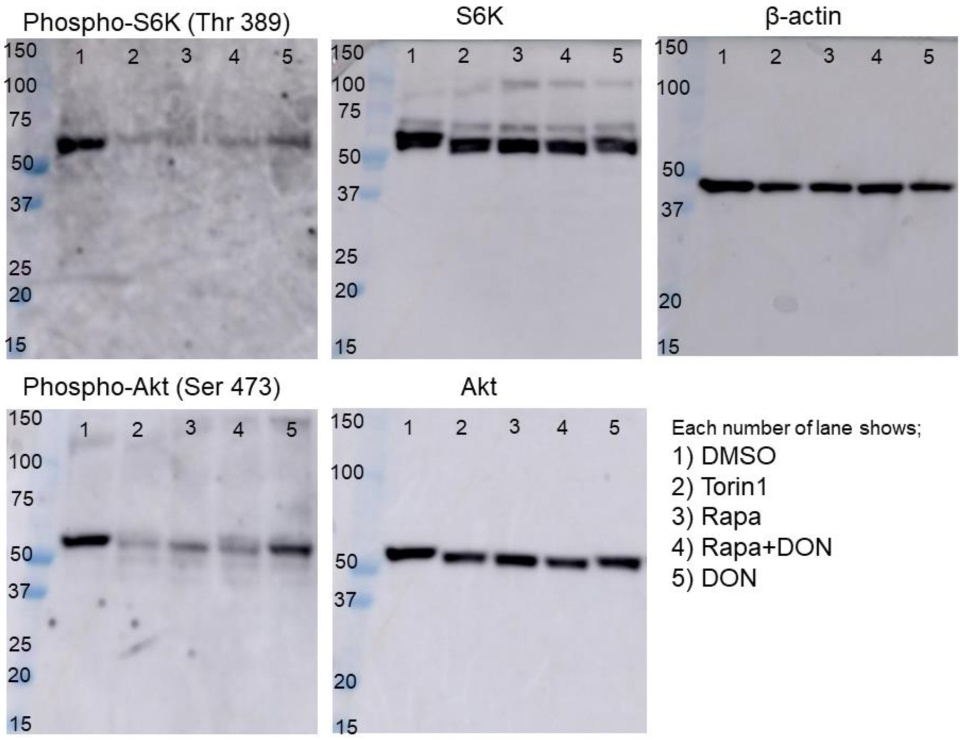

Suppl Figure 4. CFSE-labeled CD4⁺ T cells isolated from untreated Balb/c mice were cultured with several concentrations of rapamycin in combination with 5μM DON. DMSO was the positive control without DON nor rapamycin. (A) After 3 days of culture, the collected cells were assessed the CFSE fluorescence using flow cytometry. Data are representative of 4 independent experiments. One-way ANOVA with Dunnett's multiple comparison test compared to DON was performed. Bars show mean ± SEM. **P < .01; ***P < .001. (B)

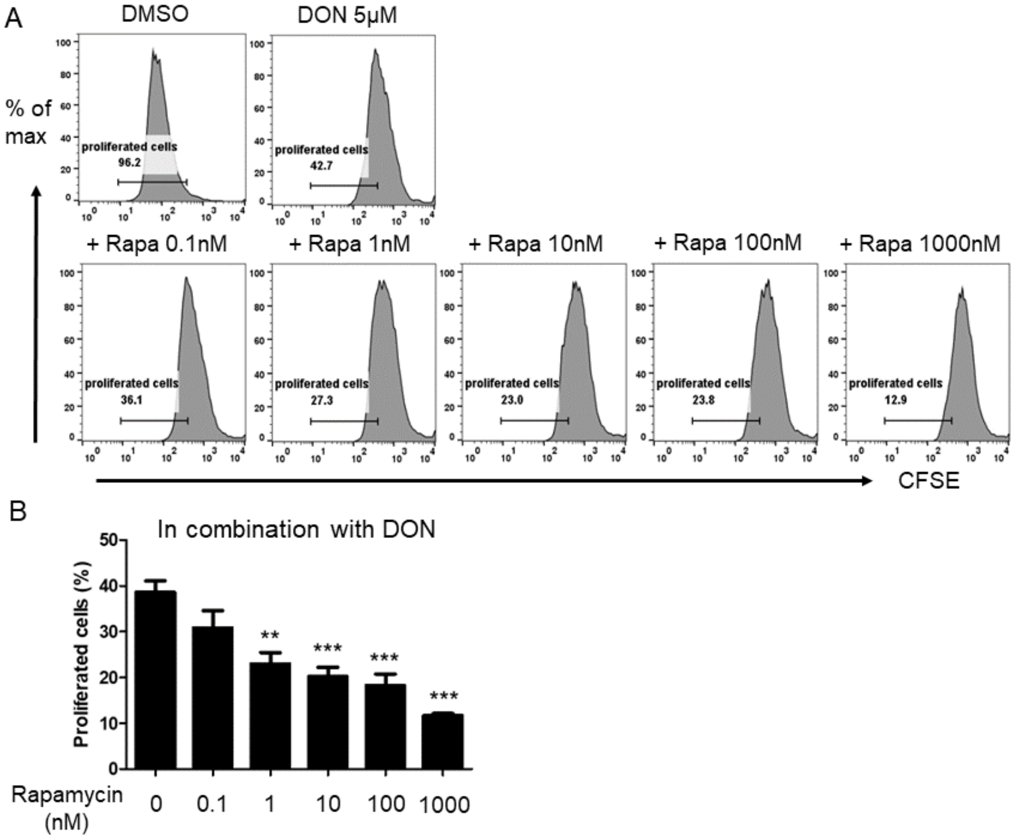

Suppl Figure 5. *In vitro*–generated G-MDSCs cultured with DSMO, rapamycin, DON, or rapamycin and DON were isolated by manual MACS against Ly6G+. The collected cells were stained with anti-CD11b and anti-Ly6G antibodies. The purity of the *in vitro*–generated G-MDSCs was analyzed by flow cytometry.

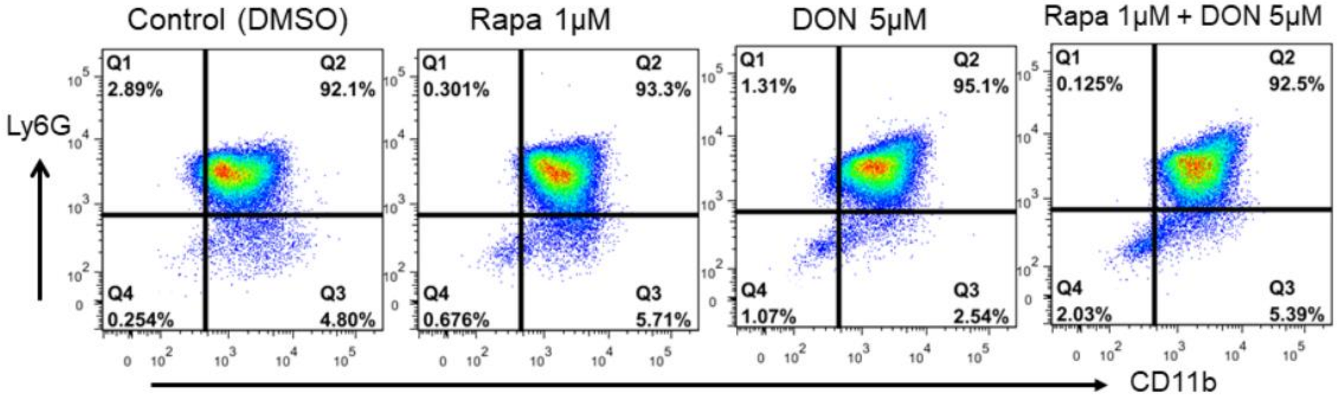

Suppl Figure 6. Body weight of mice treated with vehicle (n=6); rapamycin (Rapa, n=7), DON (n=5), or rapamycin plus DON (Rapa+DON; n=7), recorded up to day 62 after ZyA injection.

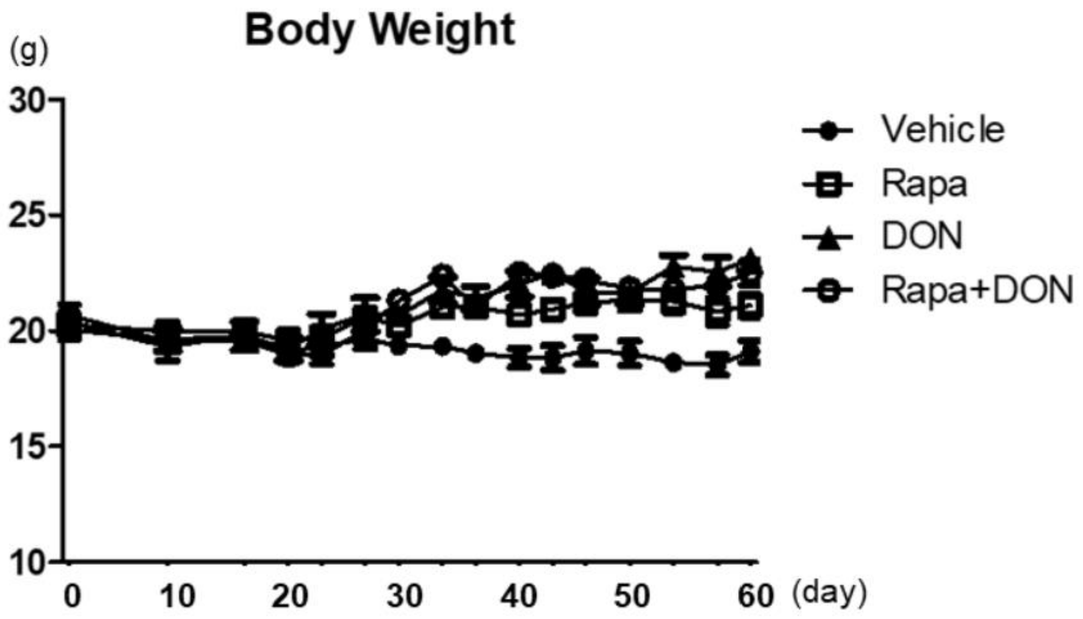

Suppl Figure 7. Immunohistochemistry in the paws of SKG mice treated with each drug regimen to measure the presence of synoviocytes (stained with cadherin-11; A), myeloid cells (stained with CD11b; B), and T lymphocyte (stained with CD3; C) was performed. Pictures are representatives of each group; original magnification 40× and the selected area was one of three areas assessed in magnification 400× (surrounded by yellow line). The extents of the presence of these cell populations by using semi-quantitative assessment (SQA). Bars show mean ± SEM. (D)

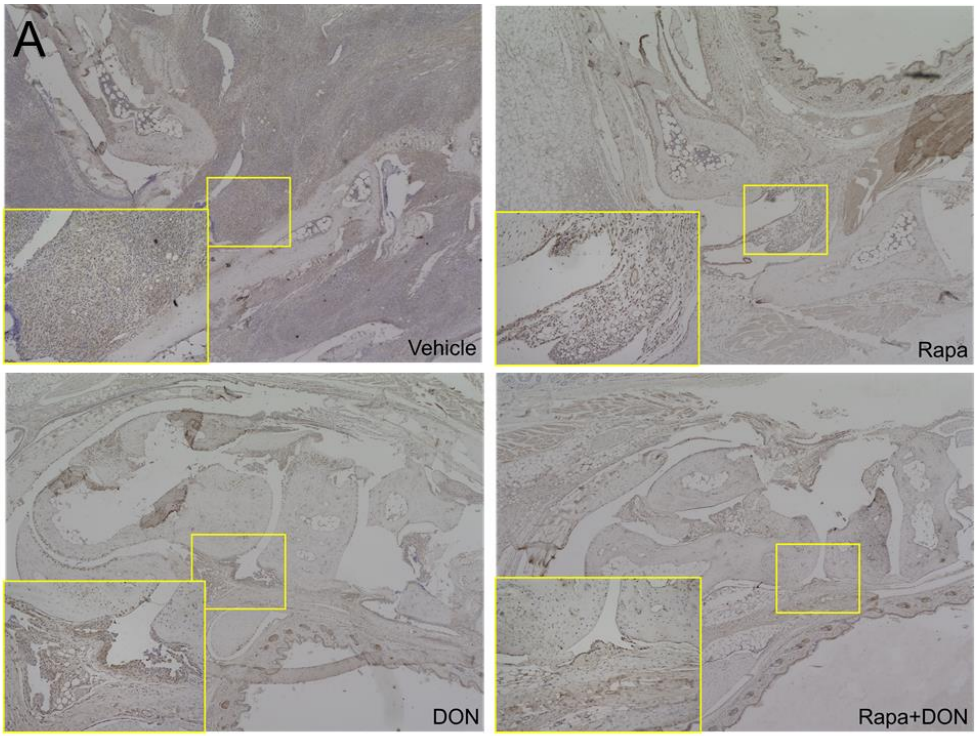

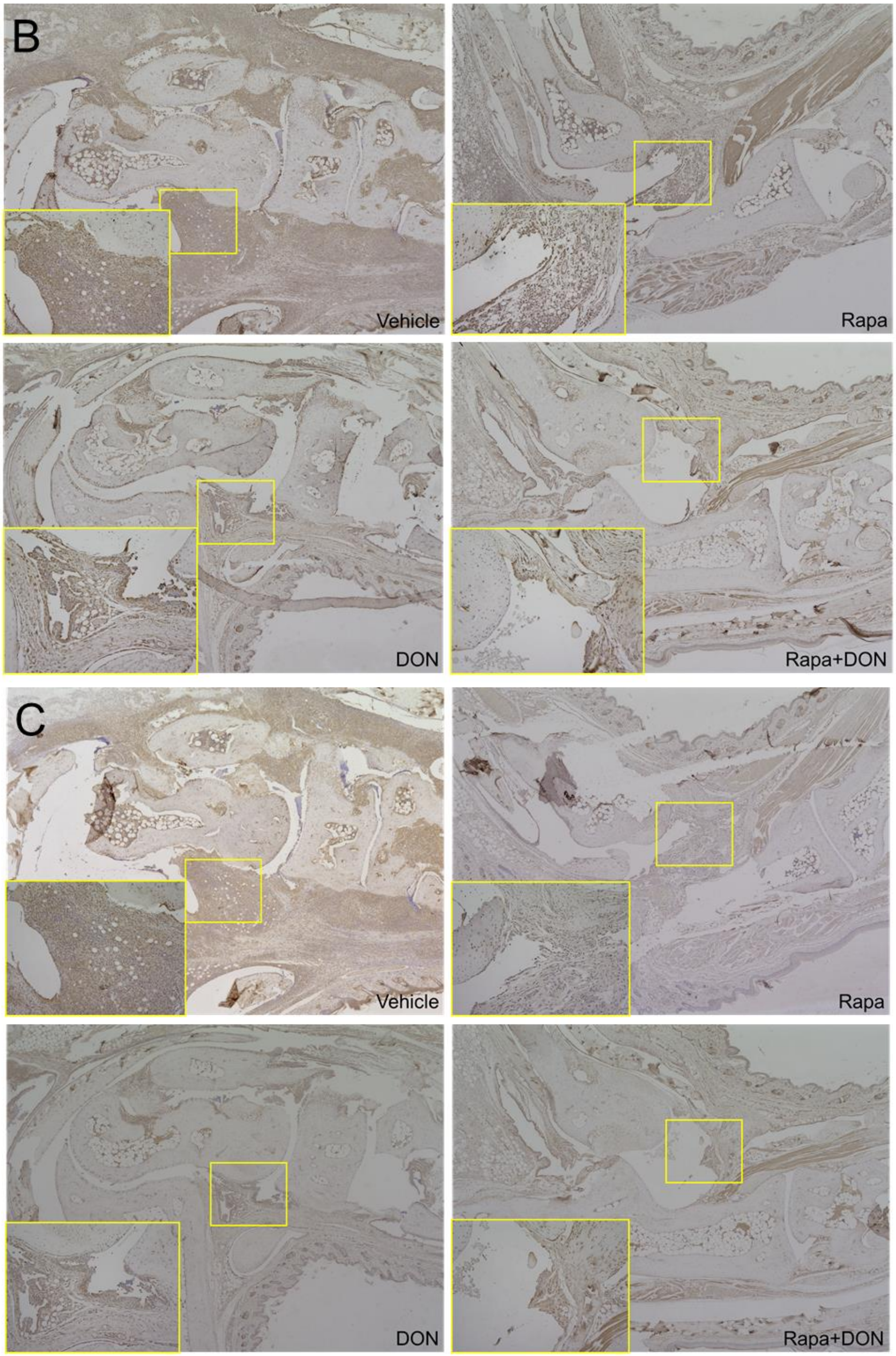
B
Vehicle
Rapa
DON
Rapa+DON
C
Vehicle
Rapa
DON
Rapa+DON

D

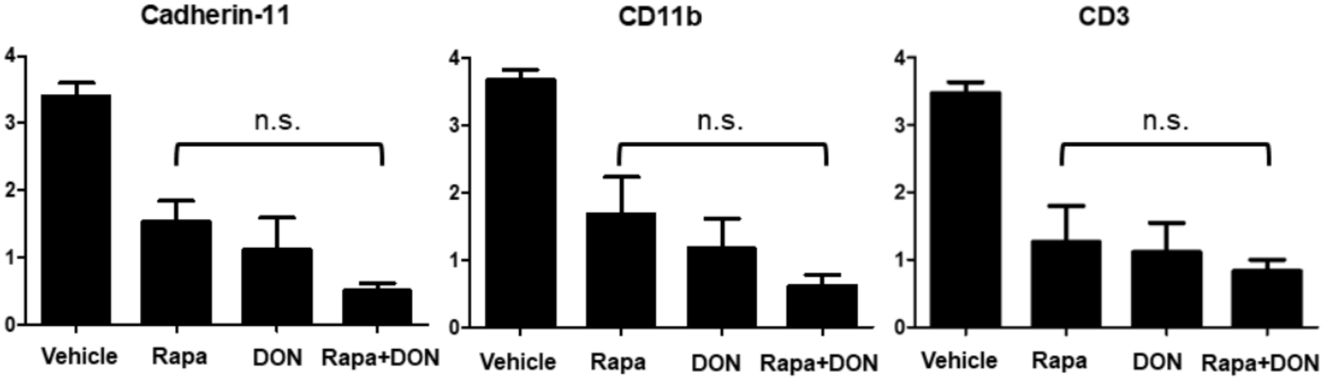
Cadherin-11
CD11b
CD3
n.s.
n.s.
n.s.
0
1
2
3
4
Vehicle
Rapa
DON
Rapa+DON
